# Carbon-Related Defects as a Source for the Enhancement of Yellow Luminescence of Unintentionally Doped GaN

**DOI:** 10.3390/nano8090744

**Published:** 2018-09-19

**Authors:** Feng Liang, Degang Zhao, Desheng Jiang, Zongshun Liu, Jianjun Zhu, Ping Chen, Jing Yang, Shuangtao Liu, Yao Xing, Liqun Zhang, Mo Li, Yuantao Zhang, Guotong Du

**Affiliations:** 1 State Key Laboratory of Integrated Optoelectronics, Institute of Semiconductors, Chinese Academy of Science, Beijing 100083, China; liangfeng13@semi.ac.cn (F.L.); dsjiang@semi.ac.cn (D.J.); zsliu@semi.ac.cn (Z.L.); jjzhu@semi.ac.cn (J.Z.); pchen@semi.ac.cn (P.C.); yangjing333@semi.ac.cn (J.Y.); lst7713@semi.ac.cn (S.L.); xingyao@semi.ac.cn (Y.X.); 2 University of Chinese Academy of Sciences, Beijing 100049, China; 3 Suzhou Institute of Nano-tech and Nano-bionics, Chinese Academy of Sciences, Suzhou 215123, China; lqzhang2012@sinano.ac.cn; 4 Microsystem & Terahertz Research Center, Chinese Academy of Engineering Physics, Chengdu 610200, China; limo@mtrc.ac.cn; 5 State Key Laboratory on Integrated Optoelectronics, College of Electronic Science and Engineering, Jilin University, Changchun 130023, China; zhangyt@jlu.edu.cn (Y.Z.); dugt@jlu.edu.cn (G.D.)

**Keywords:** unintentionally doped GaN, yellow luminescence band, carbon impurity

## Abstract

Yellow luminescence (YL) of unintentionally doped GaN (u-GaN) peaking at about 2.2 eV has been investigated for decades, but its origin still remains controversial. In this study, ten u-GaN samples grown via metalorganic chemical vapor deposition (MOCVD) are investigated. It is observed from the room temperature (RT) photoluminescence (PL) measurements that the YL band is enhanced in the PL spectra of those samples if their MOCVD growth is carried out with a decrease of pressure, temperature, or flow rate of NH_3_. Furthermore, a strong dependence of YL band intensity on the carbon concentration is found by secondary ion mass spectroscopy (SIMS) measurements, demonstrating that the increased carbon-related defects in these samples are responsible for the enhancement of the YL band.

## 1. Introduction

GaN-based third-generation semiconductor materials, including InN, GaN, AlN and their alloys have attracted extensive attention owing to their broad applications of GaN-based photonic and electronic devices, such as light-emitting diodes (LEDs) [1,2], laser diodes (LDs) [3,4,5], photodetectors (PDs) [3], and high electron mobility transistors (HEMTs) [6,7,8]. There are many defects in GaN-based materials, and these defects can weaken the performance of photonic devices by forming non-radiative recombination centers or cause serious degeneration of electronic devices by introducing extra current leakage. For unintentionally doped GaN (u-GaN) film, its typical photoluminescence (PL) spectrum consists of a near-band-edge emission (UVL) with peak intensity at around 3.4 eV and a yellow luminescence (YL) band peaking at about 2.2 eV. The origin of this YL band has been investigated for decades by experiments or theoretical calculation. However, there is also a dispute on which defect causes this YL band from GaN, although there is a popular view that this YL band is related to the deep-level defect at about 1 eV above the valence band [9,10,11]. The origin of the YL band may be quite complicated, such as vacancies, doping, or some defect complexes. Hitherto, there are two main views on the origins of this YL band among the previously found extensive reports. On one hand, this YL band is attributed to the gallium vacancy (V_Ga_) [12,13,14]. On the other hand, it is also reported that this YL band is caused by the carbon-related defects or its complex [15,16,17].

It is necessary to take a further and systematical investigation to confirm which defect should be responsible for the enhancement of the YL band emitted from GaN. Firstly, according to the previous findings [18,19], it is reported that there is also a YL band or a BL band in n-GaN and p-GaN, thus investigating the YL band in u-GaN could provide a way to further evaluate the impurity related luminescence of n-GaN and p-GaN. Secondly, the investigation on the mechanism of the YL band in u-GaN can be helpful for studying the impurity and defect related luminescence in III-nitrides, such as InGaN, AlGaN and InAlGaN. Lastly, it can provide a method of decreasing the impurity related luminescence and thus, can help to improve the performance of GaN-based optoelectronic devices, i.e., LEDs and LD. Therefore, it is necessary to investigate the origin of the YL band in u-GaN or the reason for its intensity variation. In this study, ten unintentionally doped GaN samples (u-GaN) grown under different conditions (e.g., three sets of u-GaN samples) were investigated systematically by the room temperature PL and secondary ion mass spectroscopy (SIMS). It was found that the YL band intensity has a strong dependence on the carbon concentration, demonstrating that the enhancement of the YL band from u-GaN should be ascribed to the increase of carbon-related defects. It suggests that reducing the carbon impurity through increasing growth pressure, temperature and flow rate of NH_3_ is a good way to decrease the YL band.

## 2. Materials and Methods

**Figure 1:**
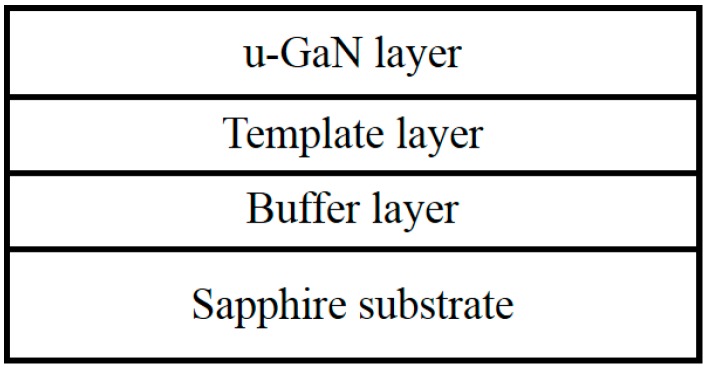
Schematic diagram of the epitaxial structure of u-GaN samples.

In this work, ten u-GaN samples are grown on c-plane sapphire substrates by metal organic chemical vapor deposition (MOCVD, Aixtron, German), and the trimethylgallium (TMGa) and NH_3_ are used as the Ga and N sources, respectively. The epitaxial structure of u-GaN is shown in Figure 1, consisting of a 20 nm thick buffer layer, a 1 μm template layer and a u-GaN layer. The growth conditions of these layers are the same for ten samples, except for the last u-GaN layer. There are three sets of u-GaN samples, and their growth conditions are listed in Table 1. First, four u-GaN samples are grown under different pressure, i.e., 200, 100, 75 and 50 Torr, which is labeled as P1, P2, P3 and P4, respectively. Second, four u-GaN samples are grown under different temperature, i.e., 1110, 1050, 1020 and 1000 °C, which are labeled as T1, T2, T3 and T4, respectively. Third, four u-GaN samples are grown under different NH_3_ flow rate, i.e., 6, 3, 2 and 1 L/min, which are labeled as F1, F2, F3 and F4, respectively. In these samples, the growth conditions of samples P1 and T4 are the same as of samples T1 and F2, respectively.

**Figure 2:**
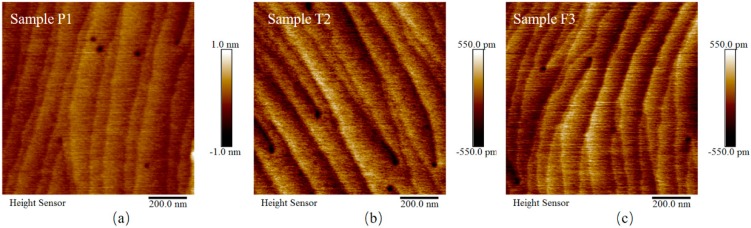
Atomic force microscopy images of samples P1, T2 and F3 in a 1 μm × 1 μm regions.

The room temperature (RT) photoluminescence measurements of these ten u-GaN samples are carried out with the 325 nm line of a He-Cd laser at an excitation density of about 0.4 W/cm^2^, and the luminescence intensity is normalized by the near-band-edge emission (marked as UVL in Figure 2a) luminescence intensity. Meanwhile, to verify how the growth conditions affect the PL intensity, SIMS measurements of these ten u-GaN samples are taken to check the concentration profiles of hydrogen, carbon and oxygen impurities, i.e., [H], [C] and [O]. The depth profiles of these impurities in u-GaN layers were measured by secondary ion mass spectroscopy (ATOMIKA 4500, Oberschleißheim, Germany) with Cs^+^ ions as the primary source, and the raster size is 80 µm × 80 µm and the collected area (in diameter) is 30 µm. Moreover, the regions taken the SIMS measurement are located around the centers of our 2-inch u-GaN wafers. 

In addition, the atomic force microscopy (AFM, Bruke, Bremen, Germany) measurements of samples P1, T2 and F3 are taken to check the morphology of our u-GaN samples. As shown in Figure 2, in 1 μm × 1 μm regions of samples P1, T2 and F3, the surface is smooth and the root-mean-square (RMS) roughness is 0.14 nm, 0.14 nm and 0.13 nm, respectively. In addition, the step flow can be seen clearly in the surface topography of these three samples. These images clearly indicate that our u-GaN samples have excellent surface topography although the thickness is as large as 1 μm. Moreover, to exclude the influence of defect density level on the luminescence of u-GaN samples, we check the defect densities of the screw and edge dislocations by X-ray diffraction (XRD, Rigaku SmartLab 3KW, Tokyo, Janpan) measurements of (002) and (102) ω-2θ rocking curves. The XRD measurements are taken with Cu Kα1 radiation, and the working current and voltage of XRD measurement is 30 mA and 40 kV. It is found that the defect densities of our ten u-GaN samples are indeed at a similar level, i.e., around 9.0 × 108 cm^−2^. Thus, combined with the depth profiles of these impurities in these samples, the influence of growth pressure, temperature and flow rate of NH3 on the PL from u-GaN will be discussed in turn below.

## 3. Results and Discussion

**Figure 3:**
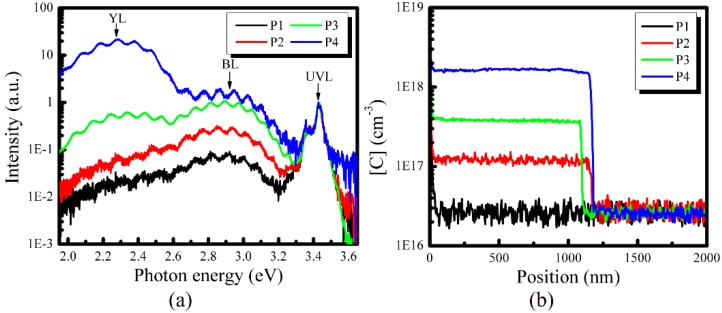
(**a**) Room-temperature PL spectra of u-GaN samples P1–P4 grown under different pressure, and (**b**) the carbon distribution profile. The arrows indicate the main luminescence bands.

First, for the samples P1–P4 grown under 200, 100, 75 and 50 Torr, respectively, the RT PL and carbon impurity distribution profiles are shown in Figure 3b. Three distinct emission bands are observed from Figure 3a, i.e., near-band-edge emission (UVL), blue luminescence (BL) band and yellow luminescence (YL) band, their peak energy is around 3.4, 2.9 and 2.3 eV, respectively. It is found that the BL intensity and YL intensity increase significantly when growth pressure decreases from 200 Torr to 50 Torr, and they become larger than the UVL intensity when the growth pressure is reduced to 50 Torr. On the other hand, the SIMS measurements show that [C] in samples P1–P4 is 2.6 × 10^16^ cm^−3^, 1.2 × 10^17^ cm^−3^, 3.7 × 10^17^ cm^−3^ and 1.6 × 10^18^ cm^−3^, respectively. It means that [C] increases by nearly two orders of magnitude when the growth pressure decreases from 200 Torr to 50 Torr. Meanwhile, it should be mentioned that SIMS results of oxygen and hydrogen impurities (not shown here) also demonstrate that [O] in samples P1–P4 is the same, i.e., 6.0 × 10^16^ cm^−3^. Compared with the pressure-related change of [C], the [H] shows less of a change, varying from 2.5 × 10^17^ cm^−3^ to 4.8 × 10^17^ cm^−3^.

Especially, the YL intensity of sample P4 is much larger than those of samples P1–P3, and it is found that the relative intensity of YL band is also much larger than the BL and UVL in sample P4. Meanwhile, [C] of sample P4 is much larger than those of samples P1–P4. It clearly shows, therefore, a strong relation between YL intensity and carbon concentration. Thus, combining the results of PL spectra with carbon distribution of samples P1–P4, it is obvious that the YL intensity or BL intensity increases when the carbon concentration increases. 

**Figure 4:**
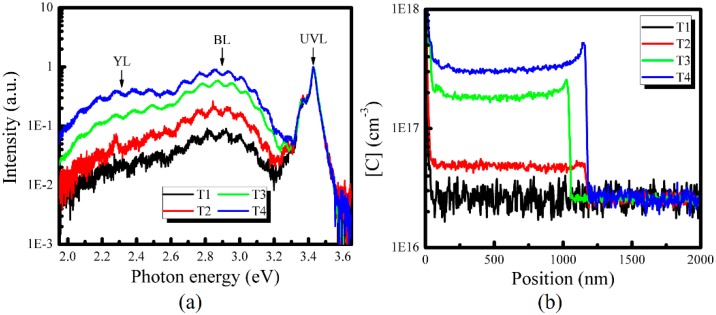
(**a**) Room-temperature PL spectra of u-GaN samples T1–T4 grown under different temperature, and (**b**) the carbon distribution profile. The arrows indicate the main luminescence bands.

In addition, four samples, i.e., T1–T4, are grown under 1110, 1050, 1020 and 1000 °C, respectively. Their RT PL and SIMS measurement results are shown in Figure 4a,b, respectively. It can be seen that the BL intensity and the YL band intensity increase when the growth temperature decreases from 1110 to 1000 °C, but their relative intensities are always less than the UVL. The impurity concentration in samples T1–T4 is also checked by SIMS measurements. Figure 4b shows that for samples T1–T4, [C] is 2.6 × 10^16^ cm^−3^, 4.9 × 10^16^ cm^−3^, 1.8 × 10^17^ cm^−3^ and 3.1 × 10^17^ cm^−3^, respectively. It means that [C] increases by more than one order of magnitude when the growth temperature decreases from 1110 °C to 1000 °C. Meanwhile, it should be mentioned that oxygen and hydrogen concentrations are also measured by SIMS. The result (not shown here) demonstrates that [O] in samples T1–T4 is the same, i.e., 6.0 × 10^16^ cm^−3^. In addition, compared with the change of [C], [H] shows less change varying from 2.5 × 10^17^ cm^−3^ to 4.9 × 10^17^ cm^−3^. Therefore, the PL spectra and carbon distributions of samples T1–T4 demonstrate that the YL intensity or BL intensity of u-GaN increases when [C] increases. This result indicates that the YL band of u-GaN can be enhanced when the carbon concentration increases.

**Figure 5:**
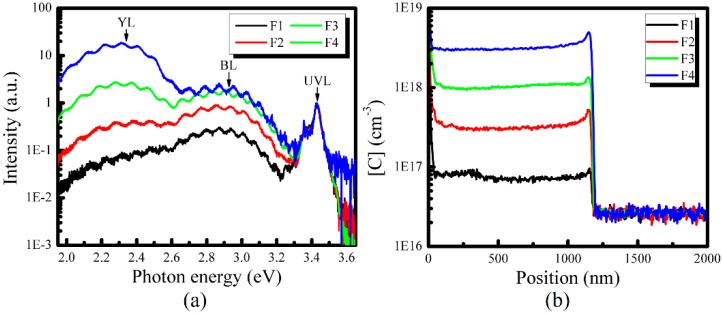
(**a**) Room-temperature PL spectra of u-GaN samples F1–F4 grown under different flow rate of NH_3_, and (**b**) the carbon distribution profile. The arrows indicate the main luminescence bands.

Moreover, we modify the carbon concentration in u-GaN furtherly by controlling the flow rate of NH_3_. The other four samples, i.e., F1–F4, are grown under the different flow rate of NH_3_, i.e., 6 L/min and 3 L/min, 2 L/min and 1 L/min, respectively. Their RT PL and SIMS measurement results are shown in Figure 5a,b, respectively. It can be seen that the BL and YL intensities increase when the flow rate of NH_3_ decreases from 6 L/min to 1 L/min, and they become larger than the UVL one when the flow rate of NH_3_ is equal to or less than 2 L/min. Meanwhile, we check the impurity concentration in samples F1–F4 by SIMS measurements. Figure 5b shows that [C] in samples F1–F4 is 8.0 × 10^16^ cm^−3^, 3.1 × 10^17^ cm^−3^, 1.0 × 10^18^ cm^−3^ and 3.1 × 10^18^ cm^−3^, respectively. It means that [C] increases by 12.5 times when the flow rate of NH_3_ decreases from 6 L/min to 1 L/min. Besides, it should be mentioned that SIMS results of O and H impurities (not shown here) also show that [O] in samples F1–F4 is the same, i.e., 6.0 × 10^16^ cm^−3^, and the [H] varies from 2.4 × 10^17^ cm^−3^ to 7.5 × 10^17^ cm^−3^. Compared with the change of [C] in samples F1–F4, [H] shows a much less change. Thus, combining the PL spectra with carbon distribution in samples F1–F4, it is clear that the YL intensity or BL intensity from u-GaN increases when [C] increases. This result reconfirms that increasing the carbon-related defects can cause the enhancement of the YL band of u-GaN.

**Figure 6:**
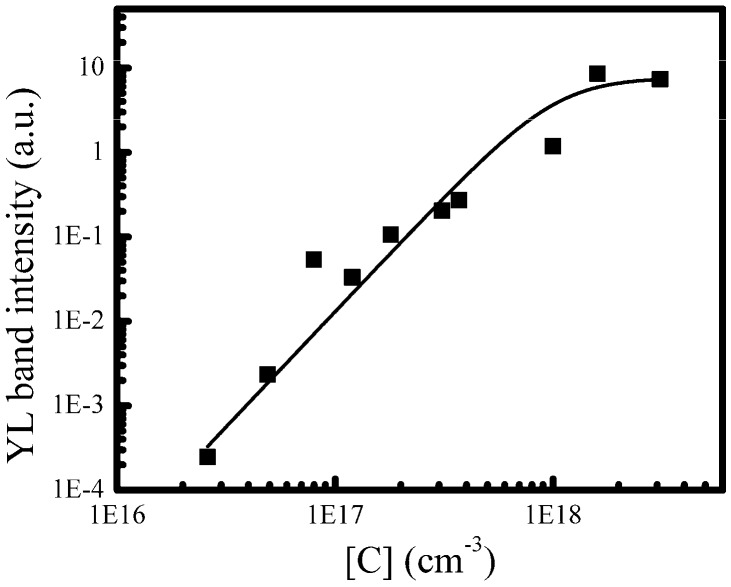
Dependence of YL band of ten u-GaN samples on the carbon concentration. The black line is used to declare the trends.

**Figure 7:**
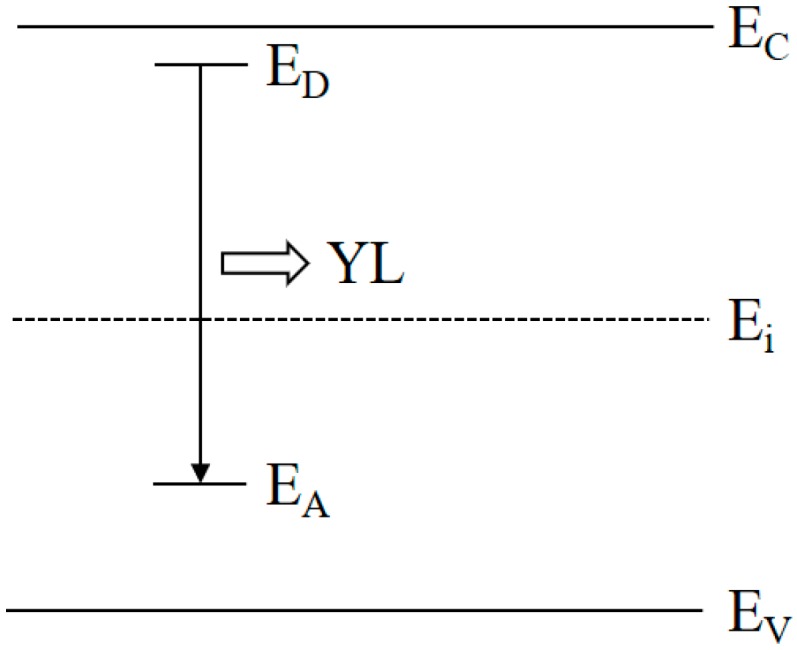
Schematic diagram of the proposed transition model about the YL in u-GaN samples.

Finally, the dependence of the integral intensity of the YL band of these ten u-GaN samples on the carbon concentrations is summarized in Figure 6. It can be seen that the integral intensity of YL band increases almost five orders of magnitude when the [C] increases about two orders of magnitude (e.g., from 2.6 × 10^16^ to 3.1 × 10^18^ cm^−3^). It demonstrates that the enhancement of the YL band of u-GaN is caused by the increase of carbon-related defects. Moreover, Figure 7 shows a proposed transition model about the YL band in our u-GaN samples. It suggests that the YL band is caused by the electron-hole recombination between the donor-related energy level (E_D_) and the acceptor (e.g., carbon related acceptor) energy level (E_A_). That is why the YL band can be enhanced by increasing the concentration of carbon impurity while reducing the growth pressure, temperature or the flow rate of NH_3_. Previous studies have reported that carbon-related defects, such as the carbon atom substituting for a nitrogen site (C_N_), the complex defect of C_N_ and O_N_ (C_N_-O_N_), the complex of V_Ga_ and C_N_ (V_Ga_-C_N_), are attributed to the origin of the YL band of GaN [15,16,17]. Meanwhile, recent theoretical calculation using ab initio density functional theory also explains how carbon-related defects cause YL band [20]. From their point of view, both the C_N_ transition between “−” and “0” charged levels and the C_N_-O_N_ transition between “0” and “+” charged levels result in the YL band. In addition, according to the SIMS measurements, the oxygen concentration of our ten u-GaN samples is the same, i.e., 6.0 × 10^16^ cm^−3^, which perhaps might suggest that YL band may originate from C_N_. Nonetheless, the dependence of YL band on the carbon concentration declares that the enhancement of the YL band of our u-GaN is attributed to the increase of carbon-related defect, and the carbon concentration in GaN can be effectively modified by the growth parameters of pressure, temperature and NH_3_ flow rate.

Moreover, in Figure 3, Figure 4 and Figure 5, it seems that the BL band is trending to increase along with increasing carbon concentration. This result is consistent with the report of R. Armitage [14], in which the blue band is observed in undoped semi-insulating GaN and intentionally C-doped GaN and BL band are attributed to carbon. Meanwhile, J. Mäkelä et al. reported the presence of deep states up to 1 eV above the valence-band maximum and the model of C_N_-related blue emission photoluminescence in Mg-doped Al_0.5_Ga_0.5_N [21]. In fact, the origin of the BL band of GaN is still in dispute. On one hand, Li et al. suggested that the BL band is the transition from the free electron to acceptor levels through double-crystal X-ray diffraction and PL measurements [22]. On the other hand, Reshchikov et al. proposed that the BL band was attributed to a V_Ga_-related complex [23], such as V_Ga_-O_N_ complex whose energy level was at 0.8 eV above the valence-band edge [24]. Recently, Demchenko et al. demonstrated that the BL band is related to a hydrogen-carbon defect complex, either C_N_O_N_-H or C_N_-H according to their hybrid functional calculations [25]. Thus, the origin of the BL band of u-GaN will be discussed in the next step, and that is why Figure 6 does not show the transition model of the BL band.

## 4. Conclusions

In summary, three sets of u-GaN samples with different carbon concentrations are grown by controlling the growth pressure, temperature and flow rate of NH_3_. They have been investigated by RT PL and SIMS. The strong dependence of YL band of u-GaN on the carbon concentration demonstrates that an enhancement of the YL band is attributed to the increase of the carbon-related defects, and the carbon concentration in u-GaN can be effectively modified by the growth parameters of pressure, temperature and NH_3_ flow rate. It provides a way to improve the performance of GaN-based optoelectronic devices by reducing the defect densities and related YL luminescence in various devices such as GaN-based LEDs and LDs.

## Figures and Tables

**Table 1:** Growth conditions of u-GaN samples. [C]: concentration of carbon impurity.

Sample	Pressure (Torr)	Temperature (°C)	NH_3_ (L/min)	[C] (cm^−3^)
P1/T1	200	1110	3	2.6 × 10^16^
P2	100	1110	3	1.2 × 10^17^
P3	75	1110	3	3.7 × 10^17^
P4	50	1110	3	1.6 × 10^18^
T2	200	1050	3	4.9 × 10^16^
T3	200	1020	3	1.8 × 10^17^
T4/F2	200	1000	3	3.1 × 10^17^
F1	200	1000	6	8.0 × 10^16^
F3	200	1000	2	1.0 × 10^18^
F4	200	1000	1	3.1 × 10^18^
